# Liquid-Liquid Phase Separation: Unraveling the Enigma of Biomolecular Condensates in Microbial Cells

**DOI:** 10.3389/fmicb.2021.751880

**Published:** 2021-10-25

**Authors:** Zixu Gao, Wenchang Zhang, Runlei Chang, Susu Zhang, Guiwen Yang, Guoyan Zhao

**Affiliations:** College of Life Science, Shandong Normal University, Jinan, China

**Keywords:** liquid-liquid phase separation, biomolecular condensates, membraneless organelles, multivalent interactions, crowded environments, cellular noise

## Abstract

Numerous examples of microbial phase-separated biomolecular condensates have now been identified following advances in fluorescence imaging and single molecule microscopy technologies. The structure, function, and potential applications of these microbial condensates are currently receiving a great deal of attention. By neatly compartmentalizing proteins and their interactors in membrane-less organizations while maintaining free communication between these macromolecules and the external environment, microbial cells are able to achieve enhanced metabolic efficiency. Typically, these condensates also possess the ability to rapidly adapt to internal and external changes. The biological functions of several phase-separated condensates in small bacterial cells show evolutionary convergence with the biological functions of their eukaryotic paralogs. Artificial microbial membrane-less organelles are being constructed with application prospects in biocatalysis, biosynthesis, and biomedicine. In this review, we provide an overview of currently known biomolecular condensates driven by liquid-liquid phase separation (LLPS) in microbial cells, and we elaborate on their biogenesis mechanisms and biological functions. Additionally, we highlight the major challenges and future research prospects in studying microbial LLPS.

## Introduction

Recent developments in the field of liquid-liquid phase separation (LLPS) have led to a transformation in our understanding of the biogenesis of subcellular membrane-less compartments. As more and more phase-separated condensates are being discovered, there is considerable interest in exploring key factors (proteins) involved in the organizations and physiological functions of the compartments. However, we are still at an early stage of understanding the precise regulation and the biochemical processes inside the condensates, lacking a global view of the interactions within/among the compartments (and the environment).

For many years, the field of compartmentalization was limited to the study of membrane-bound organelles. The presence of these functionally and structurally distinct compartments is the essential feature of eukaryotic cells. In the 1980s, small granules that behaved as fluid droplets were identified in the cytosol, and these droplets were observed to fuse together into larger assemblies known as non-membranous organelles (Strome and Wood, 1982). High-resolution imaging studies (and other methods of determining molecular composition) have revealed that membrane-less compartments generally exhibit similar dynamics and similar assembly pathways, although their position, composition, and function may differ (Mitrea et al., 2018). P granules, a type of membrane-less compartment found in *Caenorhabditis elegans*, were the first biomolecular condensate observed to form via LLPS (Brangwynne et al., 2009). These early observations concerning P bodies greatly promoted the development of this field, furthering our understanding of the physical processes driving the formation of organelles. Subsequently, evidence was provided demonstrating the involvement of LLPS in the formation of additional membrane-less organelles, including nuclear Cajal bodies (in plant and animal cells, Frey et al., 1999; Sleeman et al., 2011; Riback et al., 2020) or the homologous nucleolar body (in budding yeast, Verheggen et al., 2001), nuclear speckles (Cotto et al., 1997; Chiodi et al., 2000; Brangwynne et al., 2011; Spector and Lamond, 2011; Tripathi et al., 2012), stress granules (Buchan and Parker, 2009; Kato et al., 2012; Youn et al., 2019; Yang P. et al., 2020), and the carboxysome (a well-studied subcellular compartment in cyanobacteria responsible for sequestering and concentrating Rubisco enzymes for CO_2_ fixation) (Wang et al., 2019). Moreover, evidence was presented that LLPS may be involved in forming bacterial inclusion bodies (IBs) (Baneyx and Mujacic, 2004; Singh and Panda, 2005; Sabate et al., 2010; Chebotareva et al., 2013; Azaldegui et al., 2021; Su et al., 2021). Although membrane-less organelles have no enclosing membrane, the condensates have been demonstrated to maintain (for hours to days) stable, coherent structures capable of compartmentalizing and concentrating specific sets of molecules and exchanging material with surrounding components (Shin and Brangwynne, 2017).

An understanding of the principles underlying the formation and function of biomolecular condensates is vital for any in-depth investigation of the physiology and pathophysiology of biological processes and systems. Using up-to-date imaging, structural, and computational methods, scientists have been able to study the features of LLPS *in vitro* and *in vivo* (Alberti et al., 2018; Bracha et al., 2018; Mitrea et al., 2018). However, an in-depth study of LLPS in the comparatively small prokaryotic cell remains technically challenging, limiting our understanding of the molecular basis and biological function of compartmentalization in microorganisms. Nevertheless, recent developments in this field have yielded an extraordinary leap in understanding. In this review, we highlight representative examples of phase-separated condensates observed in microbial cells. Using these examples, we summarize the underlying mechanisms accounting for the composition, function, and the assembly/disassembly of microbial membrane-less compartments. We have also highlighted a series of challenges and future perspectives in this exciting area.

## Liquid Phase-Separated Organelles in Microorganisms

In Tiebackx (1911) firstly reported that coacervation was achieved through liquid-liquid phase separation (LLPS). Then, the concept of LLPS was applied in organic chemistry, especially in polymer chemistry (Jong and Kruyt, 1929). In this context, coacervation can be attained using either a mixture of oppositely charged polyelectrolytes (complex coacervation) or a polymer capable of self-association (self-coacervation) (Jong and Kruyt, 1929; Gabryelczyk et al., 2019). When a homogeneous polymer solution of macromolecules undergoes LLPS, two different phases are formed, a phase of concentrated molecules (dense phase) and a dilute molecule-depleted phase (dilute phase). The dense phase resembles liquid droplets (Alberti et al., 2019), and molecules in this phase can move quickly and are free to exchange interactions with multiple other molecules. Because molecules in the dense phase are highly likely to experience random molecule-molecule collisions, the potential for these molecules to complete biochemical reactions is high.

**FIGURE 1:**
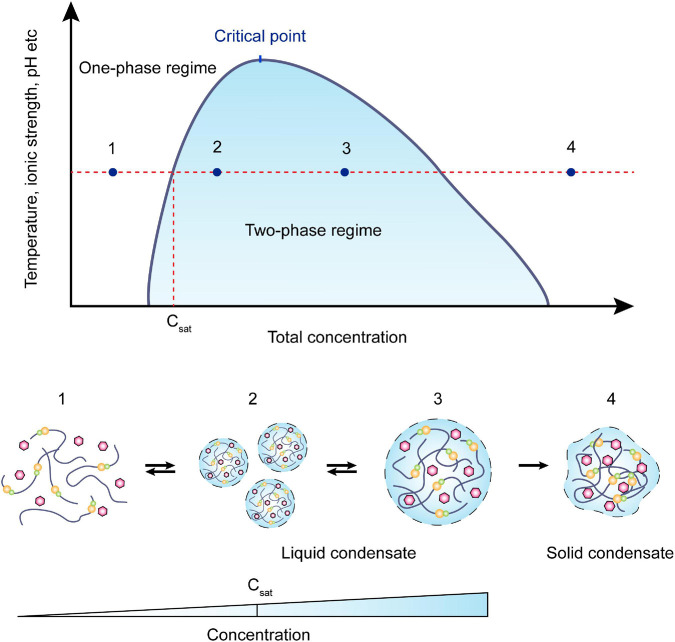
Schematic view of a phase diagram. Phase separation is a function of molecular concentration under environmental conditions such as temperature, ionic strength, pH, etc. At a concentration below *Csat*, the system remains in the one-phase regime. As the concentration increases, two-phase regimes will coexist in the system, and the required concentration is effected by the environmental change as represented in the y-axis. Within the coexistence line (black), molecules often condense into smaller droplets and fuse into bigger droplets to lower the surface tension. These processes are usually reversible. When the concentration continuously increases, the droplets may irreversibly turn into gel-like or solid condensates.

As early as 120 years ago, Wilson raised that protoplasm might be constructed by condensed liquid-droplet-like granules (Wilson, 1899). However, the commonly held view considered the cytoplasm as a fluid-like homogeneous mix of soluble proteins and compounds. It is only in the past decade, studies revealed that the cytosol does not act simply as a continuous medium but demonstrates complex rheological characteristics (Brangwynne et al., 2009; Guo et al., 2014; Shin and Brangwynne, 2017). The bacterial cytoplasm displays properties characteristic of glass-forming liquids and can solidify to resemble soft glass, depending on the metabolism, component sizes, and non-steric interactions (Parry Bradley et al., 2014; Xiang et al., 2021). Modern microscopy techniques reveal that the many proteins in bacteria tend to form large complexes targeted to specific regions within the cytosol (Abbondanzieri and Meyer, 2019). Some of the regions exhibit remarkable liquid droplet-like behaviors and undergo rapid assembly and disassembly in response to stress or cell signaling events (Sehgal et al., 2020). Evidence for this phenomenon includes the gathering of RNA degradosomes into bacterial ribonucleoprotein bodies (BR-bodies) displaying liquid-like behavior in *Escherichia coli*, *Bacillus subtilis*, and *Caulobacter crescentus* (Al-Husini et al., 2018, 2020; Hamouche et al., 2020). According to Hyman’s hypothesis (proposed by Hyman et al., 2014), the formation of phase-separated condensates in eukaryotes occurs through three main steps (Hyman et al., 2014; Strom et al., 2017; Peng and Weber, 2019): nucleation; rearrangement; and supersaturation (Figure 1). A saturation concentration, C_sat_, was defined such that: for C < C_sat_, the molecules are diffuse in solution; and for C > C_sat_, dense droplets form. If the concentration consistently increases, the liquid-like condensate may change into its gel-like or solid states (Figure 1; Nandana and Schrader, 2021). Notably, C_sat_ values are not fixed, but vary with the concentration of condensate components (Dzuricky et al., 2020; Riback et al., 2020; Zhang et al., 2021). In yeast P bodies, seven proteins are present at high concentrations (5–15 mM), forming the “core” of the condensate, and 24 additional P-body proteins are present at lower concentrations (<2.6 mM) (Xing et al., 2020). It is important to note that Hyman’s hypothesis is not limited to eukaryotic cells (Azaldegui et al., 2021). It can also be applied to bacteria, which were once considered amorphous “bags of enzymes” lacking membrane-bound organelles (Al-Husini et al., 2018). For example, *E. coli* FtsZ, a well-studied tubulin homolog that is essential for cytokinesis, is capable of forming crowding-induced condensates (Monterroso et al., 2019). *In vitro* experiments indicate that FtsZ-rich droplets are formed only when FtsZ is in a complex with nucleoid-associated inhibitor SlmA (which antagonizes FtsZ polymerization while binding to specific sites on the *E. coli* chromosome), and that concentrations of SlmA greater than 40 μM (far above the physiological concentration) are required for FtsZ condensates to form (Han et al., 2012).

Although the fundamental role played by phase separation in the spatiotemporal organization of essential microbial processes has recently been revealed (Table 1 and Supplementary Table 1), many of these processes have been scarcely explored in bacteria, largely owing to their small sizes and to resolution limits. Nevertheless, ten bacterial LLPS systems have already been identified (Azaldegui et al., 2021), and the number is increasing. These observations support the proposal that LLPS in microbes may be more the rule than the exception. In *E. coli*, aggregated proteins can be disaggregated during environmental stresses by chaperones, and their spatio-temporal localization is changed in the process (Winkler et al., 2010). In rod-shaped bacterial cells, cell poles are special regions for the localization of signaling and sensing proteins, and here proteins like MreB exhibit random movement (Lopian et al., 2010; Van Teeffelen et al., 2011; Govindarajan et al., 2013; Shi et al., 2018). Furthermore, a high-throughput tagging pipeline of *C. crescentus* proteins revealed 153 proteins with patchy or spotty subcellular localization patterns (Werner et al., 2009). Together, the above observations provide evidence that LLPS may be widely involved in subcellular organization across different microorganisms, although compartments remain to be discovered.

**TABLE 1:** Proposed phase-separated biomolecular condensates in microbial cells.

**Systems**	**Representative species**	**Biological processes**	**Functions**	**Molecular mechanisms**	**References**
**LLPS systems in eukaryotic microbes**					
P body	*Saccharomyces cerevisiae*	Regulate gene transcription	Act against stresses	Defined modular domains (Modules), Intrinsically disordered regions (IDRs)	Decker and Parker, 2012; Jain and Parker, 2013; Hubstenberger et al., 2017; Loll-Krippleber and Brown, 2017; Luo et al., 2018
Stress granules	*Saccharomyces cerevisiae*	Regulate translation	Act against stresses	IDRs	Buchan et al., 2011; Kato et al., 2012; Jain et al., 2016; Khong et al., 2017
Large 1 (Lge1) protein	*Saccharomyces cerevisiae*	Accelerate the ubiquitination of histone	Regulate metabolic flux	IDRs	Turco et al., 2015; Kim et al., 2018; Gallego et al., 2020
G body	*Saccharomyces cerevisiae*	Enhance glycolysis	Act against stresses	IDRs	Jin et al., 2017; Fuller et al., 2020
Pyrenoid	*Chlamydomonas reinhardtii*	CO_2_ concentration	Regulate metabolic flux	IDRs	Mackinder et al., 2016; Freeman Rosenzweig et al., 2017; Wunder et al., 2018; He et al., 2020
Yeast ataxin-2 protein (Pbp1)	*Saccharomyces cerevisiae*	Regulate cellular signaling and autophagy	Act against stresses	Modules	Kato et al., 2019; Yang et al., 2019
DNA repair droplet	*Saccharomyces cerevisiae*	DNA repair	Act against stresses	IDRs	Lao et al., 2008; Oshidari et al., 2020
Membrane invagination	*Saccharomyces cerevisiae*	Endocytosis	Act against stresses	IDRs	Bergeron-Sandoval et al., 2021; Lyon et al., 2021
Prion protein	*Saccharomyces cerevisiae*	Regulate translation	Act against stresses	Modules, IDRs	Franzmann et al., 2018
Heterochromatin protein 1 (HP1)	*Schizosaccharomyces pombe*	Chromatin compaction	Regulate metabolic flux	Modules	Canzio et al., 2013; Larson et al., 2017; Sanulli et al., 2019
TBP associated factor 14 (Taf14)	*Saccharomyces cerevisiae*	Regulate gene transcription	Regulate metabolic flux	Modules	Schulze et al., 2010; Chen et al., 2020; Peil et al., 2020
Cajal body homologs	*Saccharomyces cerevisiae*	Telomerase recruitment	Regulate metabolic flux	Modules	Verheggen et al., 2001; Mao et al., 2011
**LLPS systems in prokaryotic microbes**					
Carboxysome	*Synechococcus elongatus*	CO_2_ concentration	Regulate metabolic flux	IDRs	Cameron et al., 2013; Chen et al., 2013; Sun et al., 2019; MacCready et al., 2020; Oltrogge et al., 2020
BR-bodies	*Caulobacter crescentus*	Regulate RNA metabolism	Act against stresses	IDRs	Hardwick et al., 2011; Al-Husini et al., 2018, 2020; Bayas et al., 2018
ParABS DNA segregation system	*Escherichia coli*	Regulate DNA segregation	Regulate metabolic flux	Modules	Sengupta et al., 2010; Graham et al., 2014; Sanchez et al., 2015; Debaugny et al., 2018; Guilhas et al., 2020
RNA polymerase clusters	*Escherichia coli*	Control transcription	Regulate metabolic flux	Modules, IDRs	Cabrera and Jin, 2003; Weng et al., 2019; Ladouceur et al., 2020
Pole-organizing protein (PopZ)	*Caulobacter crescentus*	Control spatial patterning	Regulate metabolic flux	IDRs	Dahlberg et al., 2020; Lasker et al., 2020
Single-stranded DNA-binding protein (SSB)	*Escherichia coli*	DNA replication, repair, and recombination	Act against stresses	Modules, IDRs, Crowded environments	Zhao et al., 2019; Harami et al., 2020
ATP-binding cassette transporter (Rv1747)	*Mycobacterium tuberculosis*	Cell growth	Regulate metabolic flux	IDRs	Spivey et al., 2011; Heinkel et al., 2019; Owen and Shewmaker, 2019
Filamentous temperature-sensitive protein Z (FtsZ) assembly	*Escherichia coli*	Cell division	Regulate metabolic flux	Crowded Environments	Monterroso et al., 2016, 2019
PolyP granules	*Pseudomonas aeruginosa*	Starvation response and regulation of DNA replication	Act against stresses	Modules, IDRs, Crowded environments	Kreuzer, 2013; Racki et al., 2017
DNA-binding protein from starved cells (Dps)	*Escherichia coli*	Protect nucleoid from damage	Act against stresses	IDRs	Kim et al., 2004; Janissen et al., 2018

## Control of Phase Separation in the Formation of Biomolecular Condensates

Although many key questions regarding the organizing principle and physicochemical driving forces of phase separation remain unanswered, in many cases, weak and reversible multivalent interactions between proteins and/or nucleic acids have been demonstrated to be important drivers of biomolecular condensates (Banani et al., 2017). Several different theories explaining phase separation in condensates have been proposed, positing a role for electrostatic interactions, cation-π interactions, aromatic interactions, volume exclusion/crowding, surface tension, or the permeability rate of molecules. With these theoretical frameworks, it may now be possible to explain how the assembly, composition, dynamics, physical properties, and biochemical functions of these biomolecular condensates are regulated. Here, we focus on known mechanisms involved in driving LLPS in microorganisms.

### Multivalency-Driven Phase Separation

**FIGURE 2:**
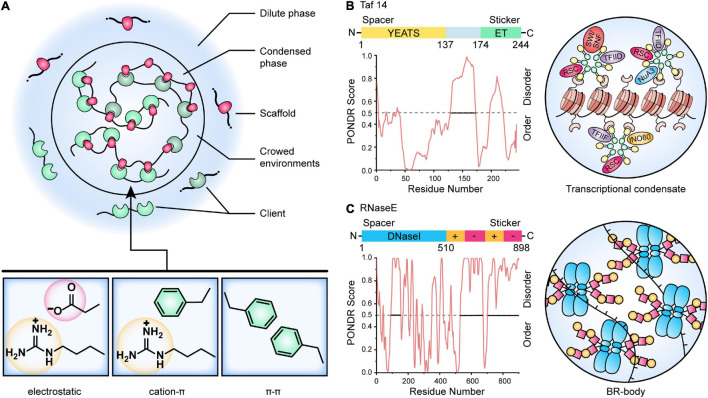
A model for the control of biomolecular condensates. **(A)** Multivalent interactions that drive LLPS. Scaffold molecules (red) that undergo LLPS are in stoichiometric excess (often in a crowding environment) and enriched for defined modular domains or intrinsically disordered regions. Client molecules (green) are recruited by binding to the free cognate sites in the scaffold. The critical scaffold/client or scaffold/scaffold interactions include electrostatic, cation-π, and π-π contacts. **(B)** Model of yeast Taf14-mediated transcriptional condensate. The Taf14 protein contains two main domains, an N-terminal YEATS (Yaf9, ENL, AF9, Taf14, Sas5) domain (yellow) that recognizes lysine acylation modification, as well as a C-terminal ET domain (green) that is reported as a protein-protein interaction domain and recognizes peptide substrates. The disordered regions of Taf14 were predicted by PONDR (Xue et al., 2010). Taf14 works as a scaffold protein that promotes phase separation of condensates and concentrates different transcriptional machinery to form Taf14-containing complexes, thereby enhancing transcription efficiency (Chen et al., 2020). **(C)** Model of *Caulobacter* RNase E BR-body assembly. The domain architecture for the RNase E protein is shown, and the disordered regions were predicted by PONDR (Xue et al., 2010). The N-terminal catalytic DNaseI domain (blue) and C-terminal disordered regions (yellow and red) are highlighted. The disordered regions contain positive-charged patches (Arg-rich RNA binding sites, yellow) and negative-charged patches (facilitating multivalent interactions with RNA, red), causing self-assembly of BR-bodies into condensates through electrostatic interactions (Al-Husini et al., 2018, 2020).

In Li et al. (2012) proposed that multivalent interactions are key factors involved in the phase separation of biomolecules. This view holds that biomolecular condensates are composed of large numbers of multivalent molecules, and thus they contain a variety of elements that control intramolecular or intermolecular interactions. For example, complex condensations can be built through the processes that receptors use to specifically combine with ligands. Therefore, increasing the number, valence, and interaction force of receptors and ligands may promote the formation of stable and large cell condensations. If these interactions occur among multivalent molecules, the molecules will form oligomers and condensations with large stoichiometric ratios (Jin et al., 2019). The essential proteins that drive reversible condensate formation are classified as “scaffolds,” and proteins that preferentially partition into the condensates have been classified as “clients” (Figure 2A). Notably, the two roles are not static or absolute, and it can be hard to unambiguously distinguish these roles in some biomolecular condensates under set environmental conditions (Banani et al., 2016, 2017). In cells, the diffusion speed of clients is much faster than that of scaffolds, and thus client/scaffold interactions are more transient than scaffold/scaffold interactions. The interactions are therefore often selective. For instance, bacterial polar protein PopZ has been shown to act as a selective scaffold that imposes a diffusion barrier to cytosolic proteins (such as the signaling protein CtrA) and constrains the mobility of these proteins at cell poles (Lasker et al., 2020). Furthermore, these interactions are frequently promoted by proteins composed of multiple-folded modular domains and/or intrinsically disordered regions (IDRs) (Gomes and Shorter, 2019).

### Proteins With Defined Modular Domains

Proteins with defined modular folding domains can assemble into higher-order oligomers via intermolecular interactions involving other proteins harboring compatible modular domains. These intermolecular interacting modular domains may be comprised of multiple folded domains or short linear motifs. A typical example from microorganisms is *E. coli* NusA, a transcription anti-termination factor that interacts directly with RNA polymerase (RNAP) (Ladouceur et al., 2020). NusA, working as a scaffold, contains six folded domains, including two C-terminal acidic repeat Arg-rich domains that recruit clients such as RNAP (and other anti-termination factors). After the scaffold has been built (scaffold proteins have been assembled), more molecules can be recruited to the system to complete the assembly of the condensates. The folded modular domains are often connected by IDRs or low complexity regions (LCRs), and these determine the material properties of the condensates (Harmon et al., 2017). TATA-binding protein-associated associated factor 14 (Taf14) from yeast was once thought as an exception that do not contain IDR or LCR (Chen et al., 2020), but sequence analysis using PONDR (Xue et al., 2010) and SMART (Letunic and Bork, 2018) show that it has two IDRs with Arg/Lys-rich and Glu-rich (Figure 2B). Taf14 is a well-studied, phase-separated transcriptional regulator that associates with a variety of other transcriptional regulators. It contains a YEATS (Yaf9, ENL, AF9, Taf14, and Sas5) domain as an effective reader of histone lysine crotonylation via a unique π–π stacking mechanism and an extra-terminal (ET) domain that recognizes a common motif in diverse transcriptional coactivator proteins such as RSC, SWI/SNF, NuA3, INO80, TFIID, and TFIIF (Andrews et al., 2016; Chen et al., 2020; Figure 2B). Meanwhile, some Taf14-binding partners (e.g., Tfg1) have a number of ET-binding sites that balance the stoichiometric ratio of different complexes in the compartmentalized transcriptional unit (Andrews et al., 2016; Chen et al., 2020).

### Proteins With Intrinsically Disordered Regions (IDRs)

In comparison to proteins with defined modular domains, proteins containing IDRs are characterized by more multi-valency and more flexible interaction modes, and therefore they represent the most abundant class of macromolecules that can drive phase separation under physiological conditions. By definition, IDRs lack a defined three-dimensional structure, and they encode multiple short-length amino acid motifs which can provide the basis for multivalent weakly adhesive intermolecular interactions. These motifs typically have a strong bias toward a limited number of amino acids, and are referred to as low complexity sequences (LCSs). They are classified as “stickers” because they demonstrate adhesive properties through π-π stacking, cation-π interactions, or charge-charge interactions (Figure 2A; Wang et al., 2018; Martin et al., 2019). Sequences between the motifs are referred to as “spacers.” Site-directed mutagenesis (or other modifications) in spacer residues can affect the thermophysical properties of the proteins, and thus change the material properties of condensates (Wang et al., 2018). A surprising degree of motional organization of IDPs (intrinsically disordered proteins) has been detected on the ps – ns scale, with IDPs demonstrating fast local vibrations and conformational sampling of backbone dihedral angles, and this may drive LLPS (Salvi et al., 2017).

Based on the examples already known, the biased amino acid compositions of IDRs in bacteria share common hallmarks with IDRs from higher eukaryotes. Thus, IDRs can be: (1) Rich in polar and uncharged amino acid residues such as Gln and Asn. Examples include the “prion-like” sequences in NIDR, the linker IDR of SARS-CoV-2 (Perdikari et al., 2020), and the Gln-rich region in McdB proteins that drives the positioning of the carboxysome (Cameron et al., 2013; MacCready et al., 2020); (2) Rich in charged residues such as Arg/Lys and Glu/Asp. Examples include the Arg-rich C-terminal domain of RNase E that is required for assembly of the core of the bacterial ribonucleoprotein body (BR-body) (Figure 2C; Al-Husini et al., 2018, 2020). Using these concepts, Wei et al. (2020) has developed an artificial membrane-less organelle in *E. coli* through heterologous overexpression of silk-like proteins using IDRs containing GGX (X = Lys, Tyr, Gln, or Ala) motifs, and this condensate is capable of catalyzing biochemical reactions (Yang et al., 2016; Wei et al., 2020).

Intrinsically disordered regions are notably scarce in bacterial proteomes (comprising less than 2–5% of the proteome) when compared with eukaryotic proteomes (where they comprise 30–40% of the human proteome) (Ward et al., 2004). However, this scarcity does not mean that phase-separation proteins are less relevant in bacteria. Indeed, evidence is accumulating to suggest that IDRs are key players within bacteria, and that these proteins drive LLPS to achieve cell divisions, metabolisms, and nucleoid organizations (Abbondanzieri and Meyer, 2019). Besides, several dedicated computational tools and resources have been published to serve as platforms for collecting, predicting, and annotating LLPS-associated proteins, providing convenient guides to study LLPS proteins in microbes. These databases include PhaSepDB (^1^You et al., 2020), LLPSDB (^2^Li et al., 2020), DrLLPS (^3^Ning et al., 2020), PhaSePro (^4^Mészáros et al., 2020), and so on. For a detailed review, see Pancsa et al. (2021).

### Crowded Environments

The cytosol is a highly crowded environment in which macromolecules such as proteins, nucleic acids, and polysaccharides must push against and compete with each other to carry out their biological functions (McGuffee and Elcock, 2010; André and Spruijt, 2020). The macromolecule concentration in the cytosol of *E. coli* has been estimated to be ∼300 – 400 mg/mL. *In vitro* studies have demonstrated that the addition of “inert” crowding agents can induce or enhance LLPS in almost all cases. These agents help mimic a system with high viscosity and a low diffusion coefficient that is favorable for biochemical reactions. Using this system, the effects of pH, temperature, and ionic strength factors can be generally explored (André and Spruijt, 2020). For example, the addition of BSA facilitated the condensation of single-stranded DNA-binding protein (SSB) (Harami et al., 2020), whereas PEG/DNA enhanced the formation of phase-separated condensates composed of FtsZ-SlmA-SBS (Monterroso et al., 2019). In most of these cases, macromolecular crowding can be conceptualized as an “excluded volume effect” (i.e., different species cannot occupy the same space). Thus, inert crowding agents exclude other species from a definite volume (the excluded volume) (Minton, 1990; Ellis, 2001). In general, the total excluded volume depends on the size of the target biomolecules, their number, and their shape (Ellis, 2001). Using the example of the formation of FtsZ-SlmA-SBS droplets, the PEG/dextran system induced an asymmetrical distribution of the condensates (Monterroso et al., 2019). In general, the exclusion volume of macromolecules (such as proteins) is much larger than that of small molecules. As a consequence of this exclusion volume, there can be an accompanying increase in the effective concentration of biomolecules of several orders of magnitude, and this may alter the equilibrium, thermodynamic, and kinetic properties of biochemical reactions (Laurent, 1963). Moreover, this can lead to the formation of biomolecular condensates (Figure 2A; André and Spruijt, 2020). Notably, investigations of crowded environment effects were mainly performed *in vitro* by mimicking cytosol conditions. However, the excluded volume is affected by the crowders’ abundance, size, and polydispersity (Kim et al., 2015; Yang D. et al., 2020). For example, a 25% decrease in the crowding level from the physiological level was proposed to lead to an utterly diffuse chromosome. In contrast, a 30% increase in the crowder level could lead to a three-fold decrease in the volume of *E. coli* nucleoids (Yang D. et al., 2020). Besides, even the most widely used uncharged crowders (such as PEG) are usually not chemically inert. They may mediate non-steric interactions that contribute to folding proteins and chromosomes *in vivo* (Sheth and Leckband, 1997; Xiang et al., 2021). Thus, it is important to investigate the crowding effect in living cells.

Remarkably, there are two issues with the organizing principles that require further consideration, “nucleation” and “nuclear size control” (Hyman et al., 2014). During nucleation, molecules that can randomly assemble with the correct configuration are able to form new droplets. However, because of the limited time, homogeneous nucleation is extremely difficult (Malinovska et al., 2013). Nucleation can occur more favorably at pre-existing locations, such as ribosomes, RNA, etc., and thus cells may control the number and configuration of nucleation. Regarding nuclear size control, cells can control the nucleus size by stopping the merging process (Feric and Brangwynne, 2013). Using the surface effect, cells can utilize additional components that can only be dissolved in droplets to prevent droplets from Ostwald ripening (Webster and Cates, 1998; Zwicker et al., 2015; Bressloff, 2020). Currently, frameworks have been proposed for studying the non-equilibrium dynamics of the dense cellular aggregates, facilitating the link between phase separation and the gene regulatory processes inside the nucleus (Yamamoto et al., 2020; Kuan et al., 2021; Laghmach and Potoyan, 2021).

## The Biological Function of Phase-Separated Condensates in Microorganisms

In cells, phase separation is controlled by the assembly and material state of a variety of chaperone proteins, posttranslational modifications (PTMs), and cellular factors, and these in turn determine the size, assembly rate, and material properties of protein condensates, ensuring that distinct cellular functions can be spatiotemporally coordinated (Wang and Zhang, 2019; Quiroz et al., 2020). The various biological activities coupled with LLPS include the classification of misfolded and unwanted proteins for degradation, chromatin organization, gene expression, the assembly of signaling clusters, actin- and microtubule-based cytoskeletal networks, the asymmetric segregation of cell fate determinants, and the formation of pre- and post-synaptic density signaling assemblies (Marrone et al., 2019; Zhang et al., 2020). The functional mechanisms of biomolecular condensates in microorganism are listed in Table 1. Here, we highlight the main functions and summarize them into two main categories.

### Regulating Metabolic Flux

#### Enhancing Activities by Concentrating Enzymes and Substrates

**FIGURE 3:**
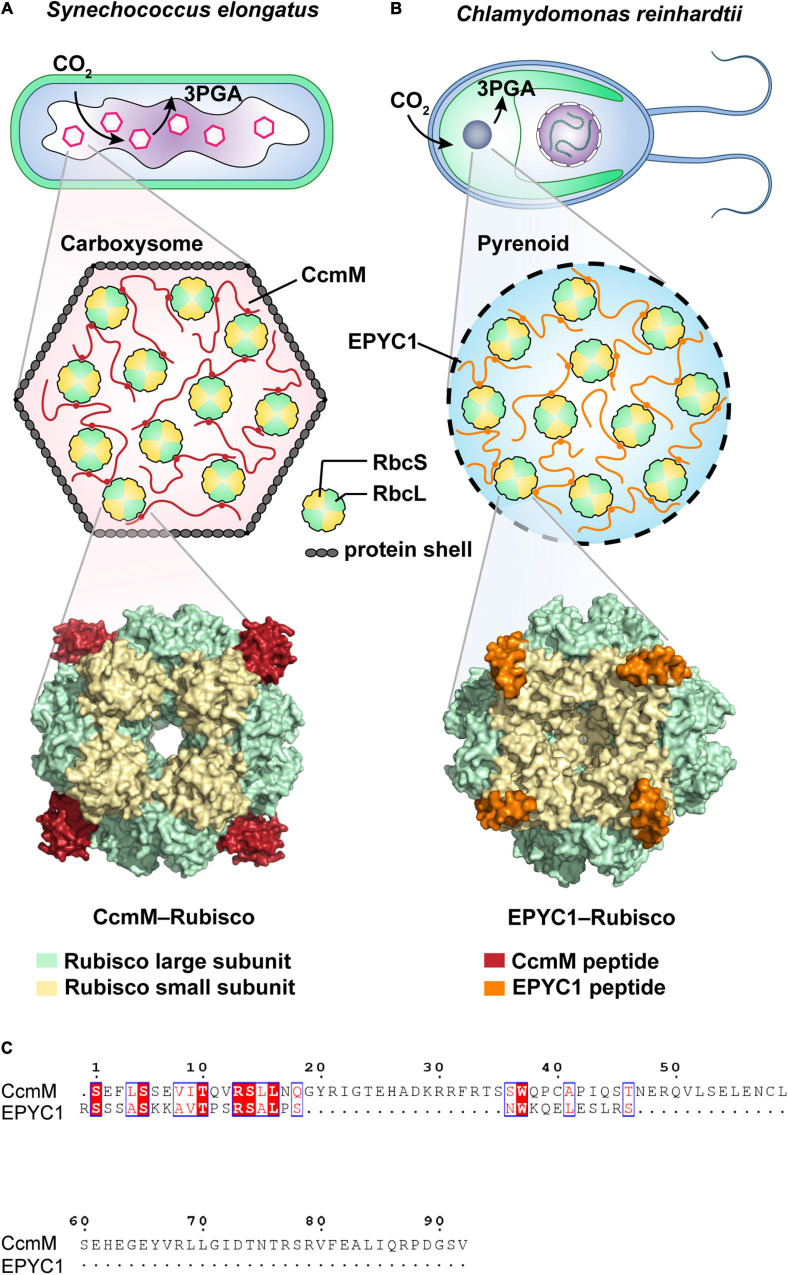
Schematic illustrations of CO_2_-fixing phase-separated liquid organelles in prokaryotic or eukaryotic cells. **(A)** Carboxysome-based Rubisco condensate found in the prokaryotic cyanobacterium *Synechococcus elongatus* PCC7942. As a scaffold protein, CcmM peptide (red) binds the Rubisco large subunit (RbcL, green) and Rubisco large subunit (RbcS, yellow) via salt bridges and van der Waals contacts to form the CcmM-Rubisco complex. It includes the condensates in the carboxysome covering with protein shells. As shown in the cryo-EM structure (6hbc, Wang et al., 2019), CcmM fills a pocket between the RbcL dimers and the loop of RbcS. **(B)** Pyrenoid-based Rubisco condensate found in the eukaryotic microalgae *Chlamydomonas reinhardtii*. The pyrenoid matrix is predominantly composed of Rubisco-EPYC1 complexes, forming by the multivalent interactions of EPYC1 peptide (orange) and Rubisco (green and yellow) (Freeman Rosenzweig et al., 2017; Wunder et al., 2018; Meyer et al., 2020; Barrett et al., 2021). Cryo-EM supported a structural model (7jsx, He et al., 2020), showing that EPYC1 binds close to the equator of the Rubisco cylinder and forms a codependent network of the specific low-affinity bonds (Mackinder et al., 2016; He et al., 2020). **(C)** Alignment of the Rubisco-binding regions from both CcmM and EYPC1 peptides by using Clustal Omega (Sievers and Higgins, 2021) and ESPript 3.0 (Robert and Gouet, 2014).

While membrane-bound organelles in eukaryotic cells are widely known to sequester biochemical pathways, membraneless organelles are also capable of organizing internal biochemical reactions. A classic example is the ribulose-1,5-bisphosphate carboxylase/oxygenase (Rubisco) condensates found in both eukaryotic and prokaryotic photosynthetic microorganisms. In cyanobacteria and other chemoautotrophic bacteria, Rubisco (the most abundant protein on the planet and the first major enzyme in the Calvin cycle) is encapsulated in a specialized protein-encased micro-compartment termed the carboxysome (Figure 3A). This compartment facilitates HCO_3_^–^ accumulation and conversion into CO_2_, known as the CO_2_ concentrating mechanism (CCM). Due to its proteinaceous shell, the carboxysome was previously believed to be para-crystalline in nature. However, recent discoveries have revealed that biogenesis of the β-carboxysome is achieved through LLPS by forming Rubisco-CcmM condensates (Figure 3A; Wang et al., 2019). In contrast, initiation of the *a*-carboxysome involves the coalescence of Rubiosco and CsoS2, a protein containing IDRs (Oltrogge et al., 2020). Furthermore, even distribution of the carboxysome is regulated by McdB, which is able to form pH-dependent droplets *in vitro* (MacCready et al., 2020).

In eukaryotic microalgae, liquid-like Rubisco-EPYC1 (Essential Pyrenoid Component 1) condensates display functional similarity to Rubisco-CcmM condensates, they are found compartmentalized in an analogous chloroplast-like CCM compartment called the pyrenoid (Figure 3B; Freeman Rosenzweig et al., 2017; Wunder et al., 2018; Barrett et al., 2021). Co-expression of EPYC1 and a plant-algal hybrid Rubisco in higher plant *Arabidopsis chloroplasts* can lead to phase-separated condensation of Rubisco in chloroplasts (Atkinson et al., 2020). Unlike carboxysome, the pyrenoid lacks proteinaceous shells. Interestingly, cryo-electron tomography (cryo-ET) revealed that the packing of the pyrenoid-based Rubisco condensates in microalgae resembles the hexagonal lattice found in cyanobacterial Rubisco condensates (Engel et al., 2015). Cryo-ET also showed that both the algal EPYC1 and cyanobacterial CcmM bind close to the equator of the Rubisco cylinder (Wang et al., 2019; He et al., 2020), although the binding sites are different. Specifically, EPYC1 binds uniquely to the Rubisco small subunit (RbcS) via electrostatic and hydrophobic interactions (Figure 3B; He et al., 2020; Meyer et al., 2020), while CcmM contacts both Rubisco large subunit (RbcL, Wang et al., 2019) and RbcS via salt bridges and van der Waals contacts (Figure 3A). Both EPYC1 and CcmM have repeat regions and intrinsically disordered proteins, and as has been side, the Rubisco condensation events appear to be regulated in a similar multivalent mechanism. However, their amino acid compositions (15.2% identity in the Rubisco-binding regions, Figure 3C) are considerably divergent (Mackinder et al., 2016; Wang et al., 2019). Altogether, these observations strongly suggest an evolutionary convergence of Rubisco condensates for the vital biological process of CO_2_ fixation. Likewise, convergent evolution has also been revealed in the formation of ribonucleoprotein (RNP) condensates aiding RNA metabolism in eukaryotic and bacterial cells, both depending on the modulation of DEAD Box ATPases. For a detailed review, see Nandana and Schrader (2021).

A phase-separated condensate may also contain multiple dense phases, so that different enzymes in the cascade can be concentrated in different compartments. In each separation phase, weak interactions between proteins and/or substrates are strongly amplified (Zeng et al., 2018), and the substrates undergo a vectorial transfer from one dense phase to another one while being enzymatically modified in each phase. The best biochemical example of vectorial organization within a biomolecule condensation is the production of ribosomes in nucleoli, where ribosomal RNA is transcribed in the innermost layer, and then processed and assembled as the ribosomal proteins pass through the outer phase (Feric et al., 2016). By concentrating one specific protein with its potential interacting molecules (and excluding other molecules), the condensates can control the specificity of the reaction. In a process akin to the classic Ostwald Ripening, larger condensates can grow bigger while smaller condensates lose molecules (Alexandrov, 2014). In *Mycobacterium tuberculosis*, ABC transporter Rv1747 undergoes controllable phase separation by acting in conjunction with several cluster-promoting factors that function as serine/threonine protein kinases (STPKs). The majority of these STPKs facilitate specific multivalent interactions by phosphorylation and Rv1747 clustering, whereas the remaining STPKs are involved in extensive signaling cross-talk and serve to dissolve the Rv1747 droplets via dephosphorylation (Heinkel et al., 2019). Thus, STPKs comprise a “multi-valency dial” which allows rapid and reversible differentiation of Rv1747 condensates in response to intracellular signaling (Glass et al., 2017; Heinkel et al., 2019).

#### Inhibition of Activities Through Sequestration

However, it should be noted that condensation does not always result in the acceleration of reaction velocity. For example, guide RNA (gRNA), the basic modification element for small nuclear RNA (snRNA), is usually concentrated in Cajal bodies. However, suppression of Cajal bodies does not appear to impact the modification effectiveness of snRNA, even though the gRNA is scattered as a consequence (Davis et al., 2015). The reasons behind the activity inhibition are manifold. Firstly, enzymes and the high concentrated scaffold proteins may interfere with each other. The scaffold may inhibit (via covalent modification) the activities of enzymes that disperse condensates (Kuznetsova et al., 2015; Banani et al., 2017). The reduction in available volume associated with high molecular condensation (molecular crowding) is also likely to influence allosteric modulation of enzymes and their binding affinity for substrates, consequently affecting enzyme activity (Kuznetsova et al., 2015). In addition, the condensates are porous structures, and the high concentration of small molecules in solution will slow down the movement of other molecules. For instance, free volume between the concentrated scaffold components may be used as a pore through which small proteins will move (as if the polymer does not exist). In contrast, the movement of macromolecules that cannot access these pores is restricted. This effect may be especially significant for condensates containing ribonucleic acid. Finally, variances in the viscoelasticity of condensates, caused by the degree of IDR maturity, the interaction dynamics of multi-domain scaffolds, RNA composition, or energy consumption processes, may affect molecular dynamics within and on the boundaries.

### Acting Against Noise and Stress

#### Buffering Cellular Noise

Liquid-liquid separation may reduce intracellular protein condensation fluctuations (protein noise) caused by the stochastic nature of gene expression in prokaryotic and eukaryotic cells. In a phase-separating system, protein concentrations inside and outside the droplets are constrained by the solubility threshold. In response to a change in the total concentration of protein, the number and size of the droplets are adjusted to reduce these fluctuations in protein concentration, thus increasing the robustness of cellular processes (Klosin et al., 2020). For example, the amount of bacterial single-stranded DNA binding protein (SSB), an essential protein in genome metabolism, is considerably higher than the amount required during replication (Bobst et al., 1985). The excess SSB and its interacting proteins are dynamically phase-separated within droplets and stored at the cell membrane. In the event of DNA damage, the droplets are rapidly (half-time, ∼70 ms) dissolved and SSB is released to protect the exposed ssDNA and repair the damage (Harami et al., 2020).

#### Sensing Stimuli and Switching

Macromolecules inside the condensates can communicate freely with external environmental factors, making it possible for the macromolecules to respond rapidly when cells sense external stimuli (Riback et al., 2017; Ruff et al., 2018). Hence, cellular functions may be turned on/off by controlling the formation or dissolution of condensates. For example, the budding yeast translation termination factor Sup35 can form reversible liquid-like condensates in response to sudden stress, ensuring that the function of the translation termination factor is retained, while the condensates can subsequently solidify to form protective protein gels. During this process, negatively charged amino acids in the prion-domain of Sup35 (which are at a high density) function as a pH sensor involved in regulating condensate formation. Upon release from stress, the gel-like condensates are dissolved (Franzmann et al., 2018). Similar processes explain the fitness advantages of yeast P-bodies, stress granules, and bacterial BR-bodies during cell stress (Shah et al., 2013; Wheeler et al., 2016; Al-Husini et al., 2018).

Another active response of condensates to stimuli involves the modulation of polymer folding states. For example, in response to heat stress, heat-labile proteins migrate into the nucleus where they bind with nucleolar protein and form condensates that protect the protein from irreversible aggregation. When the heat stress is removed, these proteins can fold into the correct conformation (Frottin et al., 2019). Likewise, mRNA poly(A) binding protein Pab1 in budding yeast undergoes rapid condensation following heat shock (Riback et al., 2017). In bacteria, similar processes were observed with the DNA-binding protein from starved cells (Dps). In response to stress, Dps binds DNA to change its topology, compacting the DNA into a dense condensate. However, RNA polymerase can freely access the “buried” genes (while other proteins are blocked) (Janissen et al., 2018). This “one-size fits all” approach protects the genome from damage and helps bacteria survive over a diverse range of stress conditions, including heat shock and oxidative stress (Karas et al., 2015; Janissen et al., 2018).

In the face of stresses, biomolecular condensates can even generate and transduce force and thus reshape the cellular architecture. A typical example in yeast is the formation of condensates at the sites of clathrin-mediated endocytosis (CME). The endocytic coat protein Sla1 at the hub of the condensates can bind with both membrane and cytosol proteins (Bergeron-Sandoval et al., 2021). By balancing condensate-membrane and condensate-cytosol interaction energies, the force is exerted sufficiently to drive membrane invagination (Bergeron-Sandoval et al., 2021; Lyon et al., 2021).

## Discussion

**FIGURE 4:**
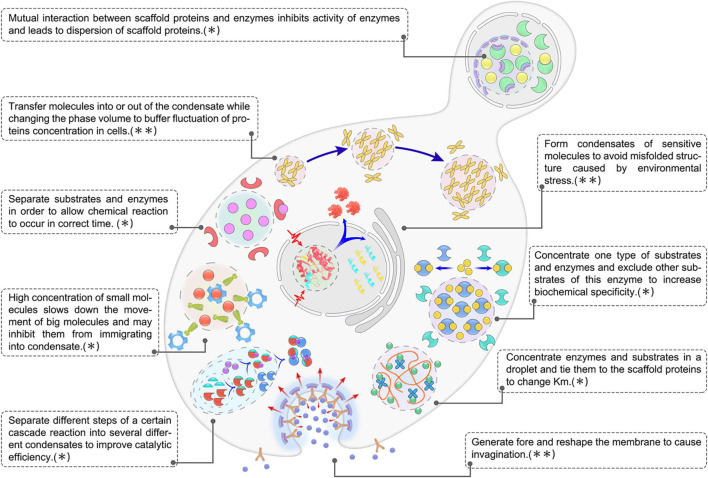
Overview of biological functions of biomolecular condensates in microbial cells. The image shown is representative of nine main functions of LLPS in microbial condensates, which could be further summarized into two categories: ^∗^,condensates play a role in regulating metabolic flux. ^∗∗^,condensates play a role in acting against noise and stress.

Evidence is accumulating that phase transitions may be a general mechanism through which microorganisms regulate cellular functions and rapidly adapt to a changing environment. The functions are presented as a model in Figure 4. However, several issues remain unresolved: What mechanisms regulate the specific recruitment of macromolecules in membrane-less organelles? In particular, why are some molecules allowed entry into these organelles while other molecules are selectively excluded? How (and under what circumstances) are these condensates assembled and disassembled? How can the biochemical reactions inside the condensates be scrutinized? By what mechanisms do some condensates divide further into additional compartments (or structured regions) that perform specialized functions? These questions may be addressed by studying the behavior of microbial cells at length over a relative long time scale, and by studying the structural, dynamic, and thermodynamic aspects of these condensates.

Numerous technical challenges need to be overcome to solve these problems. In microbial condensates (especially condensates from prokaryotic cells), the major hurdle is condensate size (Alberti et al., 2019). While traditional microscopic approaches can be used to detect LLPS in eukaryotic cells, LLPS in prokaryotic cells are typically an order of magnitude smaller (McSwiggen et al., 2019). Thus, prokaryotic condensates *in vivo* are typically <100–300 nm in diameter (Cho et al., 2018) which is beyond the spatial resolution of light microscopy (∼300 nm, Wenger et al., 2007), resulting in all the condensates appearing spherical. *In vitro* studies in simulation systems, can be a viable alternative to the *in vivo* assays. To date, almost all understanding of the protein structures and dynamics involved in bacterial condensates have been garnered from *in vitro* studies using recombinant proteins. However, these systems are comprised of only one or (at most) two components, and are considerably less complex than *in vivo* systems, which have properties that are determined by the coexistence of hundreds of thousands of macromolecules and small molecules in a highly confined volume. To mimic the crowded subcellular environment, crowding agents can be added to the *in vitro* systems. As mentioned above, however, it is not a simple matter to mimic typical condensate viscosity or viscoelasticity. Recently, single-molecule tracking/single-particle tracking (SMT/SPT) super-resolution microscopy and fluorescence correlation spectroscopy (FCS) have proved to be promising tools for investigating the properties of condensates in microorganisms (Gahlmann and Moerner, 2014; Tuson and Biteen, 2015; Sahoo et al., 2016; Jiang et al., 2017; Wei et al., 2017; Maharana et al., 2018; Sieben et al., 2018; Coelho et al., 2020; Gwosch et al., 2020; Peng et al., 2020), while proximity-dependent labeling approaches have been applied to map the protein interactome within the condensates (Govers et al., 2017; Markmiller et al., 2018; Ramanathan et al., 2018). Fluorescence recovery after photobleaching (FRAP), the “gold standard” assay in eukaryotic cells for measuring condensate fluidity and the dynamics of protein exchanges, may also be applied to study bacterial condensates, although model choice and data analysis need to be carefully considered (McSwiggen et al., 2019; Taylor et al., 2019). Together, the application of new fluorescence microscopy techniques to the study of microbial LLPS may prove ground-breaking, creating exciting new perspectives (Cambre and Aertsen, 2020).

To mimic subcellular compartmentalization and control micro-reactions in space and time, artificial membraneless organelles with liquid-like properties have been successfully constructed.

For example, artificial intracellular condensates were *de novo* designed in *E. coli* basing on a simple repeat sequence of (Gly-Arg-Gly-Asp-Ser-Pro-Tyr-Ser)XX (where XX is the number of repeats, between 20 and 80). They exhibited controllable dynamics by modulating the molecular weights (number of the repeats, Dzuricky et al., 2020). Protein/RNA coacervates, spider silk protein, and elastic-like protein were also engineered in *E. coli* with reversible formations, tunable dynamics, and selective enrichments in components, depending on the protein levels and the ratio of charged residues (Mushnikov et al., 2019; Wei et al., 2020; Yeong et al., 2020). In a recently engineered condensate comprised of small ubiquitin-like modifier (SUMOylation), enzyme activity increased approximately 36-fold in the droplets (compared with the surrounding bulk solution) (Peeples and Rosen, 2021). These studies have paved the way for the construction of synthetic membraneless organelles with designer functions in prokaryotes. These synthetic membraneless organelles have broad application prospects in biocatalysis, synthetic biology, and metabolic engineering (Deshpande et al., 2001). Multi-stimuli-responsive carriers (thermal or pH-responsive reversible coacervate droplets) can also be imbued with the ability to package and deliver drugs (Gabryelczyk et al., 2019). Furthermore, microfluidic techniques can be employed to create monodisperse coacervate droplets, making it possible to mimic diverse intracellular activities within uniform unilamellar lipid vesicles (Deshpande et al., 2001; Van Swaay et al., 2015; Deng and Huck, 2017; Love et al., 2020; Zhao et al., 2021). However, the intrinsic properties and functions of these coacervate droplets may differ dramatically as a function of size, and it remains unclear how large a condensate must grow before specific functions can arise (Lyon et al., 2021). One of the biggest challenges is spatiotemporal control over the time-programmed condensation and disassembly of the coacervate. The time-programmed phase behavior is currently available by changes in pH, temperature, ionic strength, light (UV), and more recently by enzyme-mediated catalytic activity (Shin et al., 2017; Schuster et al., 2018; Martin et al., 2019; Wheeler et al., 2019; Garabedian et al., 2021). Furthermore, by converting chemical fuels, the coacervate droplet could behave like a protocell capable of self-division, making it an ideal model for approaching the dynamic complexity of living cells (Zwicker et al., 2017; Donau et al., 2020; Karoui et al., 2021).

In summary, the number of liquid-like condensates identified in microorganisms has grown rapidly during the last few years. While the fundamental role of LLPS in membrane-less compartmentalization has drawn intense interest, new questions and hypotheses concerning the molecular mechanisms and biological processes associated with these microbial condensates have been raised. These gaps in knowledge may be filled through the development of multiscale and interdisciplinary approaches. As the field moves forward, new applications for microbial condensates will be explored.

## Author Contributions

GZ and GY contributed to conception, revision, and wrote sections of the manuscript. ZG and WZ wrote the first draft of the manuscript. GZ and ZG designed and made the figures and the table. RC and SZ wrote sections of the manuscript. All authors contributed to manuscript revision, read, and approved the submitted version.

## Conflict of Interest

The authors declare that the research was conducted in the absence of any commercial or financial relationships that could be construed as a potential conflict of interest.

## Publisher’s Note

All claims expressed in this article are solely those of the authors and do not necessarily represent those of their affiliated organizations, or those of the publisher, the editors and the reviewers. Any product that may be evaluated in this article, or claim that may be made by its manufacturer, is not guaranteed or endorsed by the publisher.

## Footnotes

1
http://db.phasep.pro/618891875618891875

2
http://bio-comp.org.cn/llpsdb/

3
http://llps.biocuckoo.cn

4
https://phasepro.elte.hu/
