# Supplementary material for: Liquid-Liquid Phase Separation: Unraveling the Enigma of Biomolecular Condensates in Microbial Cells
Source: Front Microbiol. 2021 Oct 25;12:751880. doi: 10.3389/fmicb.2021.751880 (PMC8573418; doi:10.3389/fmicb.2021.751880)
Supplement: Supplementary file 1 [file Table_1.docx]

Table S1. The thermodynamic and kinetic characteristics of membrane-less organelles. *N.D*., the precise information cannot be found.

| **Systems** | **Droplet size** | **Experimental Temperature** | **Diffusion coefficient** | **Experimental**  **Concentrate** | **References** |
| --- | --- | --- | --- | --- | --- |
| LLPS systems in eukaryotic microbes | | | | | |
| Large 1 (Lge1) protein | *N.D.* | 20°C | *N.D.* | 0.1-0.5 μM | (Kim *et al*, 2018) (Gallego *et al*, 2020) |
| Pyrenoid | *N.D.* | 40°C | *N.D.* | *N.D.* | (Wunder *et al*, 2018) (He *et al*, 2020) |
| DNA repair droplet | *N.D.* | 30°C | *N.D.* | *N.D.* | (Oshidari *et al*, 2020) |
| Heterochromatin protein 1 (HP1) | *N.D.* | 22°C | *N.D.* | 80-100 μM | (Sanulli *et al*, 2019) |
| TBP associated factor 14 (Taf14) | *N.D.* | 30°C | *N.D.* | 25 μM | (Peil *et al*, 2020) (Chen *et al*, 2020) |
| Canonical yeast prion protein (Sup35) | 3 μm (diameter) | 30°C | *N.D.* | 2 μM | (Franzmann et al, 2018) |
| LLPS systems in prokaryotic microbes | | | | | |
| Carboxysome | *N.D.* | 25°C | *N.D.* | *N.D.* | (MacCready *et al*, 2020) (Oltrogge *et al*, 2020) |
| BR-bodies | *N.D.* | 45°C | 0.0337 ± 0.0011 μm^2^ /s | 12.4 µM | (Al-Husinis *et al*, 2018) |
| ParABS  DNA segregation system | 43 ± 7 nm (diameter) | *N.D.* | *N.D.* | ~10 mM（local ParB dimer） | (Guilhas *et al*, 2020) |
| RNA polymerase clusters | *N.D.* | 37°C | 0.63 ± 0.01 μm^2^/s | 10 μM | (Ladouceur *et al*, 2020) |
| Pole-organizing protein (PopZ) | 100–200 nm (microdomain) | 28°C | *N.D.* | *N.D.* | (Lasker *et al*, 2020) |
| Single-stranded DNA-binding protein (SSB) | 5 μm (diameter) | 25°C | 0.009 ± 0.001 μm^2^/s | *N.D.* | (Harami *et al*, 2020) |
| ATP-binding cassette transporter (Rv1747) | 2600 nm^2^ (area of clusters) | 22°C | *N.D.* | 250 μM | (Heinkel *et al*, 2019) (Owen & Shewmaker, 2019) |
| PolyP granules | 200 nm (diameter) | *N.D.* | *N.D.* | *N.D.* | (Racki *et al*, 2017) |
